# Supplementary material for: Content-rich biological network constructed by mining PubMed abstracts
Source: BMC Bioinformatics. 2004 Oct 8;5:147. doi: 10.1186/1471-2105-5-147 (PMC528731; doi:10.1186/1471-2105-5-147)
Supplement: Additional File 2 — The original results of the above study (non-essential files are deleted to keep the file size under the limit set by BMC bioinformatics). [file 1471-2105-5-147-S2.bz2 › chilibotAdditionalFile2/dip10/553ID7592797E10913/html/IFNAR1_IFNA8.html]

 


 **IFNAR1** and **IFNA8** 
  
Found 13 abstracts in PubMed, retrieved 10.  
 

 What does Google say? 
 PDF only 
| .edu only 

---

- J Biol Chem, 2001   **Virus specific activation of a novel interferon regulatory factor, IRF 5, results in the induction of distinct interferon alpha **[IFNAR1]** genes.** .
  Interferon regulatory factor IRF genes encode DNA binding proteins that are involved in the innate immune response to infection.
  Two of these proteins, IRF 3 and IRF 7, serve as direct transducers of virus mediated signaling and play critical roles in the induction of type I interferon genes.
  We have now shown that another factor, IRF 5, participates in the induction of interferon A IFNA and IFNB genes and can replace the requirement for IRF 7 in the induction of IFNA genes.
  We demonstrate that, despite the functional similarity, IRF 5 possesses unique characteristics and does not have a redundant role.
  Thus, 1 activation of IRF 5 by phosphorylation is virus specific, and its in vivo association with the IFNA promoter can be detected only in cells infected with NDV, not Sendai virus, while both viruses activate IRF 3 and IRF 7, and 2 NDV infection of IRF 5 overexpressing cells preferentially induced the **IFNA8** subtype, while IFNA1 was primarily induced in IRF 7 expressing cells.
  These data indicate that multiple signaling pathways induced by infection may be differentially recognized by members of the IRF family and modulate transcription of individual IFNA genes in a virus and cell type specific manner.
